# Editorial: Perspectives in avian skeletal systems and skeletal abnormalities

**DOI:** 10.3389/fphys.2023.1229943

**Published:** 2023-06-13

**Authors:** Chongxiao Chen, Anthony Pokoo-Aikins, Shu-Cheng Huang, Prafulla Regmi, Mujeeb Ur Rehman

**Affiliations:** ^1^ Department of Poultry Science, University of Georgia, Athens, GA, United States; ^2^ Toxicology and Mycotoxin Research Unit, U.S. National Poultry Research Center, Agricultural Research Service (USDA), Athens, GA, United States; ^3^ College of Veterinary Medicine, Henan Agricultural University, Zhengzhou, China; ^4^ State Key Laboratory of Marine Resource Utilization in South China Sea, College of Oceanology, Hainan University, Haikou, Hainan, China; ^5^ Directorate Planning and Development, Livestock and Dairy Development Department, Quetta, Balochistan, Pakistan

**Keywords:** bone health, abnormalities, oxidative stress, mineral homeostasis, poultry

## Introduction

The avian skeletal system is gaining increasing attention from researchers as a unique research model for studying bone metabolism, pathologies, and the significance of bone health in relation to animal productivity and welfare. The avian skeleton is a delicate system providing structural support, minerals for eggshell formation, and even acting as an immune organ. Skeletal abnormalities in avian species can have significant implications for poultry farming. Conditions such as osteomyelitis, rickets, and chondrodystrophy, can lead to changes in gait patterns, mobility, and pathophysiologies resulting in detrimental effects on growth performance, health, and economic losses. The current Research Topic entitled “*Perspectives in avian skeletal system and skeletal abnormalities*” collected ten original research publications, including topics in bone health in meat birds (four articles), bone health in laying hens (four articles), oxidative stress and bone health (two articles). The compilation provides insights into the impact of nutrition and environmental management on bone health, the relationship between physiological parameters and bone quality, dedicated research into the mechanism of bone remodeling, and the oxidative stresses affecting bone development.

## Bone health in meat birds

The increased emphasis on genetic selection for meat production has led to birds being required to carry more muscle mass than ever, which poses significant challenges to bone health. Lameness is the most common bone issue, impacting not only birds’ performance but also animal welfare. The common causes of lameness in broilers include femoral head necrosis (FHC, or bacterial chondronecrosis with osteomyelitis, BCO) and tibial dyschondroplasia (TD).

FHC is associated with microfractures followed by opportunistic bacterial infection. According to the research from Fan et al., FHN is also linked to dyslipidemia where high lipids levels could increase the blood viscosity, leading to tissue hypoxia, elevated intraosseous pressure, and impaired nutrient transportation. These factors contribute to the development of FHN. Fan et al. investigated the lipid metabolism and bone-related parameters in healthy and spontaneous FHN broilers at 3–5 weeks of age. The results indicated a lipid metabolism disorder in FHN birds, characterized by higher total cholesterol (TC), triglycerides (TG), and low-density lipoprotein cholesterol (LDL-C) compared to healthy birds. Furthermore, metabolism-related markers provided evidence of increased fat synthesis and decomposition in the FHN group.

Femoral head separation (FHS) is characterized by the detachment of growth plate (GP) and articular cartilage. This condition predisposes the birds to FHN. The causes of FHS are still unclear, but in recent research conducted by Ibelli et al. found differential expression of 34 genes, including downregulation of chondrogenesis and bone differentiation in FHS-affected birds when compared to healthy birds. Moreover, twelve single-nucleotide polymorphisms (SNPs) associated with FHS were identified. This research project provides fundamental information to enhance our understanding of the causes of FHS.

Another common cause of lameness in broilers is tibial dyschondroplasia (TD), characterized by an abnormality in the bone growth plate where excess cartilage template remains without being replaced by bone. The diagnosis of TD typically involves invasive methods, such as exposing the tibial head to observe morphological changes in the growth plate. However, Huang et al. revealed a potential non-invasive biomarker, 4-hydroxybenzaldehyde, which can be obtained from fecal samples and used to distinguish TD broilers from normal ones. This potential marker could become a powerful tool for TD diagnosis in the field. In another research project by Lee et al., the researchers explored a different trait affecting bone quality and discovered that Japanese quails with mutated myostatin (MSTN) exhibited higher tibia bone mass and better structural quality. This finding highlights the potential application of MSTN in improving meat yield and bone quality in meat-producing birds.

## Bone health in laying hens

Laying hen bones exhibit unique structures compared to mammalian bones due to the eggshell formation process. Due the need of eggshell formation, unique woven-like medullary bones are developed during sexual maturation to provide a calcium source for eggshell. Genetic selection has led to high egg production, which constantly pressures bone renewal. As the birds age, bone quality issues become significant. Despite numerous papers published in this area, finding an effective solution to reduce this problem remains elusive. Several factors contribute to laying hen bone health, including environmental management, dietary factors, and diseases.

Regarding management, the transition to cage-free systems is a growing trend driven by animal welfare concerns and consumer preferences. A study conducted by Fu et al. demonstrated improvement in eggshell quality and bone quality in aviary systems compared to conventional cages. Notably, the femur showed increased bone resorption activity, possibly related to the enhanced eggshell quality observed. Nevertheless, it is important to note that adopting cage-free production systems can result in increased environmental impacts, a higher incidence of keel bone fractures, and increased gut health challenges. Further research is needed to assess animal welfare in cage-free systems comprehensively, determine the nutrition requirements, and develop strategies to mitigate gut health issues.

Nutrition plays an essential role in bone health and laying performance. Extensive research has been conducted to improve laying performance and bone health via nutritional interventions. Previous studies have highlighted the significance of pullet bone quality and its long-term impact on bone health and laying performance during the late laying period (Sinclair-Black et al.). Notably, White et al. conducted an interesting study demonstrating that combining 25-hydroxyvitamin D3 with optimized calcium/phosphorus levels improved pullet bone quality, as evidenced by enhanced cortical and trabecular bone 3D structures. In contrast, the research presented by Xin et al. showed that protein level (15% vs. 16.5%) and energy level (2,700 vs. 2,800 kcal/kg) during the prelay period (15–20 weeks) did not impact laying performance. Only slight improvements in egg shape index and eggshell thickness were observed in the high protein treatment group. Molecular parameters indicated that protein and energy levels influenced the expression of HPG axis-related genes of hens towards the end of the laying cycle without altering the circulating sex hormone profile.

A review by Sinclair-Black et al. summarized physiological regulations of vitamin D3, calcium, and phosphorus and provided an overview of calcium and phosphorus homeostasis during eggshell mineralization in laying hens. The review also identified several research gaps, such as FGF23 expression which is important for calcium and phosphorus homeostasis. Additionally, the lack of tools for studying avian species’ vitamin D3 metabolism and shell gland calcium transportation was highlighted. The review emphasized the limitations of applying mammalian research models to birds, given the unique characteristics of medullary bone and eggshell calcification in avian species. Therefore, additional tools, such as *in vitro* models, are needed to advance research in this area (Sinclair-Black et al.).

## Oxidative stress and bone health

Bone health issues are usually linked to nutritional imbalance, diseases, and management factors. However, research presented by Tompkins et al. has shown that coccidiosis, a protozoal disease, may contribute to bone loss by altering redox balance and impairing antioxidant status induced by *coccidia* infection caused by *Eimeria* infection. The authors of this study also mentioned that immune status could be a critical factor in the pathogenesis of bone abnormalities during intestinal parasite infections, warranting further research. This finding highlights that bones are not only structural support for birds but also vital immune organs. In another study conducted by Tompkins et al., mesenchymal stem cells (MSCs) were used as a research model to investigate the effects of oxidative stress on cell fate. The study demonstrated that long-term treatment of chicken MSCs with H_2_O_2_ impaired osteogenic differentiation. This research further emphasizes the impact of oxidative stress on bone-related cellular processes.

## Perspectives

The avian skeletal system is not only a fascinating research model for bone health but also holds great significance in enhancing food production and animal welfare. This Research Topic should inspire future projects to advance our knowledge of avian bone physiology and health. Future research in this field could focus on developing novel tools to evaluate bone quality, uncovering avian osteoimmunology, and exploring the immune functions of bones under various challenges. Furthermore, research is still needed to improve skeletal health through advancements in nutrition, environmental management, breeding strategies, and other relevant areas.

